# Mindfulness Reduces Adolescent Depression Through Stress Appraisal and Cognitive Reactivity: Evidence from a Four-Wave Longitudinal Study

**DOI:** 10.3390/medicina61071154

**Published:** 2025-06-26

**Authors:** Filipa Ćavar Mišković, Goran Milas

**Affiliations:** Institute of Social Sciences “Ivo Pilar”, 10000 Zagreb, Croatia; filipa.cavar@pilar.hr

**Keywords:** mindfulness, stress, stressful life events, depression, reappraisal, adolescents, mental health

## Abstract

*Background and Objectives:* Adolescence is a critical yet vulnerable developmental stage, characterized by increased exposure to stressful life events (SLEs), which are strongly linked to the onset and progression of depression. Although mindfulness has been consistently associated with lower depressive symptoms, the mechanisms underlying this relationship—particularly in adolescents—remain underexplored. Prior research suggests that mindfulness operates through cognitive mechanisms, such as reduced rumination, enhanced emotional regulation, and greater cognitive flexibility. However, much of this work is cross-sectional, limiting causal interpretation and often overlooking distinctions between direct and indirect effects. This study aimed to clarify two proposed pathways through which trait mindfulness may reduce depressive symptoms in adolescents: (1) a direct pathway involving core cognitive–emotional processes, and (2) an indirect pathway, where mindfulness supports more adaptive stress appraisal. A secondary objective was to assess whether these indirect effects vary across different types of stressful life events. *Materials and Methods:* We analyzed longitudinal data from 3897 adolescents (M_age = 15.9; 51.2% female) across four waves spaced approximately six months apart. Structural equation modeling (AMOS) was used to evaluate both direct and indirect effects of trait mindfulness on depression, with stress domains included in separate analyses. *Results:* Trait mindfulness was strongly negatively correlated with depression (r = –0.39 to –0.56). The direct effect of mindfulness on depression was substantial (β = –0.60 to –0.74), while indirect effects via cognitive reappraisal were smaller (β = –0.10 to –0.26 for stress reduction; up to –0.17 for depression). Indirect effects varied across stress domains and were generally modest. *Conclusions:* Mindfulness appears to reduce adolescent depressive symptoms through both direct and indirect pathways. The more pronounced direct effect likely reflects underlying mechanisms, such as reduced rumination and enhanced emotional regulation. Although weaker, the indirect pathway—mediated by more adaptive stress appraisal—adds meaningful explanatory value. Together, these findings underscore mindfulness as a key protective factor and highlight its potential for informing targeted, resilience-based interventions in adolescent mental health.

## 1. Introduction

Adolescence is a turbulent stage of development, characterized by new challenges and exposure to various stressors [1,2]. Rapid biological and social changes require significant behavioral adaptability, leaving many adolescents vulnerable to mental health difficulties [3]. Among these, depression becomes increasingly prevalent, with nearly 20% of individuals meeting the criteria for clinical depression by age 18 [4]. The consequences of adolescent depression often persist into adulthood, contributing to long-term mental health impairments [5]. Although many adolescents do not meet the full clinical criteria for a depressive disorder, a significant number still experience depressive symptoms that place them at heightened risk of developing major depression in early adulthood [4,6,7]. These young individuals are also more susceptible to a range of harmful consequences later in life, such as substance dependency, involvement in dangerous or unlawful behaviors, and a gradual decline in physical well-being [8]. Particularly concerning is the marked increase in the prevalence of high depressive symptoms among adolescents, which surged from 24% in the early 2000s to 37% within the next ten years [9]. This upward trend underscores a growing public health concern, emphasizing the urgent need for early intervention and preventative strategies.

### 1.1. Stressful Life Events and Depression

It is well established that adverse life circumstances and stressful life events (SLEs) play a critical role in the onset and progression of stress-related mental health challenges and disorders [10]. The investigation of SLEs as primary triggers for stress and stress-induced psychopathology has deep roots in psychological research, gaining significant traction more than half a century ago with the development of one of the first inventories cataloging such adversities [11]. Since then, a substantial body of evidence has consistently demonstrated a robust association between exposure to SLEs and the development of depression [12,13,14,15].

This line of inquiry is particularly vital during adolescence—a pivotal developmental period bridging childhood and emerging adulthood [16]. Adolescence is characterized by profound physical, emotional, and social transformations, which can themselves serve as significant stressors [17]. Arnett [1] characterizes this stage as inherently turbulent and emotionally volatile, often marked by mood instability, parent–child conflicts, and engagement in high-risk behaviors, such as alcohol and substance use [18]. Moreover, adolescents must navigate evolving social dynamics within their families, peer networks, and academic environments [19,20], further compounding their exposure to stress.

Consequently, adolescence constitutes a uniquely vulnerable period, with a heightened risk for the onset of depressive disorders compared with both childhood and adulthood [2,21,22,23]. Understanding the influence of SLEs during this developmental window is essential for informing targeted prevention and intervention strategies aimed at reducing the long-term psychological burden.

### 1.2. The Role of Mindfulness

Mindfulness has received considerable attention in psychological and prevention-oriented research for its potential to alleviate depressive symptoms and enhance overall mental health during adolescence [24,25], though findings warrant cautious interpretation [26].

An inverse relationship between mindfulness and both stress and depression has been found consistently across this research field. Higher trait mindfulness is consistently found to be directly associated with lower symptoms of depression and stress [27]. Higher levels of mindfulness have been closely linked to lower levels of adolescent depression and lower levels of perceived stress [28]. Rather, considering the long-term effects of mindfulness-based programs for adolescents, research shows beneficial effects on adolescents’ emotional resilience and overall mental well-being [29].

A meta-analysis [24], concerning the effects of mindfulness-based intervention on adolescent mental health, indicates beneficial and promising effects of mindfulness interventions across all considered RCTs. That is, significant positive effects of MBIs have been found for various mental health problems, such as depression, anxiety, and stress. Concerning the cognitive aspects of mindfulness effects, the same authors report the significant and positive effects of mindfulness-based interventions on executive functioning and attention [24], both important underlying cognitive mechanisms in depressed-thinking pathways.

Research concerning longitudinal mindfulness effects on adolescent depression suggests that trait mindfulness is associated with a reduced risk of developing depressive symptoms at both the between- and within-person levels [30], thus advocating for the promotive role of mindfulness-based coping programs in reducing adolescent depression.

### 1.3. Mechanisms and Pathways of Mindfulness in Coping with Stress

While MBIs show promising benefits in alleviating mental health problems [24], especially when developmentally timed, the exact mindfulness mechanisms underlying these pathways are still being detected. The beneficial efficacy of mindfulness in mitigating stress effects and facilitating coping is often attributed to its capacity to alter stress appraisal and subsequent stress response, that is—to regulate emotions.

Garland’s Mindfulness to Meaning Theory offers a comprehensive framework for understanding how mindfulness facilitates positive emotion regulation by influencing key cognitive and emotional mechanisms [31]. Central to this theory is mindfulness’s role in facilitating a cognitive shift from negative stress appraisals and fostering a metacognitive state that promotes positive reappraisal. Such cognitive decentering is proposed to reduce cognitive and subsequently behavioral reactivity, which often emerges as the primary stress response. Instead, metacognitive awareness rather promotes emotional flexibility. Through its effects in executive functioning and emotion regulation process, mindfulness modulates the neural attention–appraisal–emotion pathway, thus enhancing and regulating automatic responses toward negative (stress) stimuli [32], thus allowing individuals to observe their thoughts and feelings more objectively rather than acting upon the automatic and habitual responses. Thereby, mindfulness enables the cognitive process of decentering that interrupts the prior negative appraisal of the stressful situation and promotes cognitive capacity for reinterpretation of stressors through positive reappraisal [31]. These mechanisms suggest that mindfulness may reduce depressive symptoms and mitigate its negative effects on mental health, primarily through fostering adolescents’ positive reappraisal of stressful situations, thus buffering against reactive and ruminative cognitive patterns that maintain neural circuits in depressive symptomatology.

Furthermore, Garland et al. [32] suggest that mindfulness also promotes adaptive emotion regulation involved in executive functioning, which includes reduced activation in neural regions closely associated with rumination and habitual negative thinking, and heightened activation in the prefrontal cortex involved in cognitive control.

Therefore, considering the complex relationship between stress, depression, and mindfulness, mindfulness seems to have both indirect effects in mitigating depressive symptoms through cognitive decentering and positive reappraisal, as well as a direct effect on rumination, and subsequently reducing depressive cognitive schemas that underlay and foster this type of psychopathology.

### 1.4. The Current Study

Previous research has consistently shown that mindfulness is associated with lower levels of depression [27,28], primarily through cognitive mechanisms such as reduced rumination, increased cognitive flexibility, and improved emotional regulation [32]. However, much of this evidence comes from cross-sectional studies, which limit the ability to draw causal inferences and do not distinguish between mechanisms that directly alleviate depressive symptoms and those that influence how individuals reinterpret stressful experiences.

Mindfulness cultivates a non-judgmental awareness that can enable adolescents to reframe stressful situations more adaptively, viewing them as manageable challenges rather than overwhelming threats. The current study aims to disentangle two hypothesized pathways by which mindfulness may reduce depressive symptoms among adolescents:

1. Indirectly, through the reappraisal of stressful events, thereby diminishing their perceived threat;
2. Directly, through the modulation of cognitive processes such as rumination and emotional reactivity.

**Figure 1:**
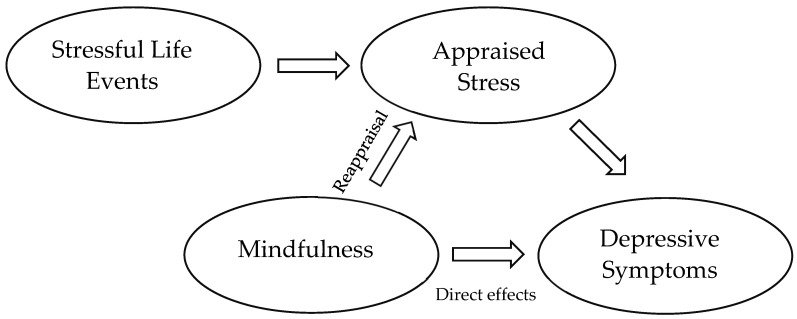
Hypothetical model of the dual pathways underlying mindfulness’s influence on depression.

Figure 1 illustrates a hypothetical model of these two pathways. The first proposed effect of mindfulness operates through the reinterpretation of stressful events, which numerous studies have identified as a key factor in the onset of depressive episodes. By reducing the perceived threat of stressors, mindfulness may lessen their impact on depression. The second hypothesized effect is direct, whereby mindfulness alleviates depression through cognitive processes such as decreased rumination and improved emotional regulation.

A secondary objective of this study is to examine the strength of the reappraisal effect across various domains of stressful events. Prior research indicates that cognitive reappraisal may be more effective for certain types of stressors, particularly those perceived as controllable or conducive to personal growth. Adolescents are more likely to reframe experiences, such as academic challenges, interpersonal conflicts, breakups, or school transitions, as opportunities for learning and self-discovery. In contrast, more severe or traumatic events—such as bereavement, abuse, or chronic illness—are less amenable to reappraisal and often require extended emotional processing and professional support. Mindfulness may play a critical role in this process by promoting emotional distance, acceptance, and cognitive flexibility, all of which facilitate more adaptive interpretations of stressful experiences. To investigate this further, we categorized common adolescent stressors into seven domains, hypothesizing that mindfulness will be most effective in reappraising controllable life events or those perceived as opportunities for personal growth, whereas its impact on less controllable stressors will be more limited.

## 2. Materials and Methods

### 2.1. Participants and Procedure

As part of the Longitudinal Adolescent Stress Study, data were gathered through a mobile application from students enrolled in 17 secondary schools across Zagreb, Croatia. The research was carried out over a two-year period, from March 2022 to February 2024. To select participating schools, a proportional random sampling method was used, taking into account the distribution of students across different institutions. Schools were further stratified based on their primary educational focus, which initially identified 18 vocational schools and 10 gymnasiums out of a total of 51 public secondary schools. Art schools were not included in the selection process, as they represent only a small fraction (approximately 3%) of the student population and often allow for dual enrollment alongside other secondary schools. This study received official approval from the relevant ministry prior to school recruitment. Of the schools contacted, 15 agreed to participate in the initial phase, with two more joining in the second wave of data collection, resulting in a final sample consisting of 10 vocational schools and 7 gymnasiums. Within each participating school, data were collected from five first-year and five second-year classes. The final total sample included 3897 adolescents (Mean age at first wave = 15.9 years, 51.2% female). Data collection was conducted in four waves at approximately six-month intervals: Wave 1 (March 2022)—2486 participants; Wave 2—1794 participants; Wave 3—2319 participants; Wave 4 (Final wave)—1575 participants. Before participating, students were briefly informed about this study’s objectives and research focus. Then, they provided informed consent, with parental or guardian consent also obtained via email for those under the age of 15.

Table 1 presents the composition of the sample across all four waves. Initially, the sample was balanced to represent the population of high school students in Zagreb in terms of gender and school type. However, in subsequent waves, the sample became increasingly skewed toward a higher proportion of female students and those attending gymnasiums. This shift can be attributed to two main factors. First, by the third wave, many students from three-year vocational programs—who were predominantly male—had already completed their education and were no longer available for follow-up testing. Second, both excused and unexcused absences were more common among students in vocational schools than in gymnasiums, which further contributed to attrition within that subgroup.

### 2.2. Measures

#### 2.2.1. Stressful Life Events

The Adolescent Inventory of Stressful Life Events (AISLE) [33] was developed as part of the STRESS LOAD project, which examined the adverse effects of stress on adolescents. The AISLE consists of 49 stressful events identified by a team of psychologists as relevant and significant for the target population (ages 14–18). Most of these events were adapted from existing inventories [34,35,36], while additional items were included to ensure comprehensive coverage. The inventory encompasses both chronic and acute stressors, ranging from daily hassles to major life events.

Stressful events were classified into the following categories:

“Health problems”: serious physical illness (e.g., chronic illness, illness requiring hospitalization, or long-term rest); serious physical injury (e.g., concussion, broken arm, broken leg…); serious mental illness or crisis; serious dissatisfaction with one’s own appearance that lasts longer than 6 months; suicidal thoughts or suicide attempt; alcohol problems; drug problems.

“Harassment or abuse”: another student in the class teased, insulted, or made fun of you; another student in the class threatened to hit you or did hit you; other students in the class excluded you from an activity; you have been a victim of sexual harassment or abuse; you were a victim of psychological harassment or abuse.

“Problems related to school”: more than 30 h of unjustified absences from school; more than 120 h of justified absences from school; bad grades in school; serious conflicts with other students; serious conflicts with teachers; warning, reprimand, or expulsion from school; repeating a class; change of school (does not include transfer from primary to secondary school).

“Problems in close relationships”: a serious argument with parents or guardians; a serious argument with friends; ending a close friendship; problems in a relationship with a boyfriend/girlfriend; mental or physical abuse in a relationship with a boyfriend/girlfriend; breaking up with a boyfriend/girlfriend; unwanted pregnancy or miscarriage.

“Loss of a close person or pet”: loss of a parent or guardian; loss of a sibling; loss of another close relative (grandfather, grandmother, uncle, aunt, uncle, aunt, nephew…); loss of a close friend; loss of a pet.

“Health problems of a close person”: serious illness or physical injury of a parent or guardian; serious illness or physical injury of a sibling; serious illness or physical injury of another close relative (grandfather, grandmother, uncle, aunt, uncle, aunt, nephew, etc.); serious illness or physical injury of a close friend; serious psychological crisis or problem of a parent or close family member; alcohol or drug problems of a parent or close family member.

“Problems related to the family situation”: frequent parental quarrels; family violence, parental neglect; problems with the law of a parent or a close family member; loss of a parent’s or guardian’s job; moving from another city or country; relocation of parents or guardians to another country for work; long-term unemployment of parents or guardians; divorce of parents or guardians; financial problems in the household (impossibility to pay off loans, basic living expenses…); present damage to the apartment or house because of an accident (flood, earthquake, fire…).

Participants indicated which stressful events they had experienced in the past year and rated their perceived stressfulness on a five-point scale (1 = Not at all stressful to 5 = Extremely stressful).

Scale scores provide two complementary but distinct measures of stress:

Objective Stress: The total number of stressful events an individual has experienced, calculated as the sum of reported events within a specific domain or across all domains.

Subjective Stress: The total perceived stress an individual has experienced, determined by summing the stressfulness ratings of experienced events within a specific domain or overall.

#### 2.2.2. Reliability Considerations

As with other stressful life event (SLE) inventories, the AISLE is not intended to demonstrate high internal consistency. In some domains, item correlations are expected (e.g., poor grades and school absences), while in others, such as the loss of a family member versus the loss of a friend, correlations are not anticipated. Consequently, alpha coefficients are presented primarily for reference rather than as definitive indicators of scale reliability. The same rationale applies to test–retest reliability, as certain SLE domains are unlikely to remain stable over time. Test–retest correlations, therefore, vary by domain, ranging from 0.38 for the loss of loved ones to 0.57 for relationship problems in the context of objective stress, with slightly higher values observed for subjective stress. The full correlation matrix of SLEs is available in the Supplement (Table S1).

#### 2.2.3. Mindfulness

Mindfulness was assessed using two measurements adopted for adolescents, both of which concern the receptiveness of everyday experiences.

#### 2.2.4. MAASA

Mindful Attention Awareness Scale for Adolescents [37], commonly used in measuring trait mindfulness in adolescents aged 14 to 18 years, consists of a total of 14 items concerning everyday experiences. Participants rate the frequency of each experience on a scale of 1 (*Almost never*) to 6 (*Almost always*). To obtain the total score, all items were first reverse-scored and then averaged. MAAS-A has shown high internal consistency (α = 0.90) and satisfactory test–retest validity (r = 0.65) in the sample of Croatian adolescents.

#### 2.2.5. CAMM

Child and Adolescent Mindfulness Measure [38], consisting of 10 items, was used as another mindfulness measure. Participants had to rate the extent of their everyday experiences (e.g., “I get upset for having feelings that don’t make sense.”) on the scale from 0 (never true) to 4 (always true). The total score was calculated as the mean of all items, following reverse scoring. Similar to MAAS-A, CAMM has shown high internal consistency (α = 0.88) and acceptable test–retest validity (r = 0.62) in the sample of Croatian adolescents (see Table 2).

#### 2.2.6. Depression

The severity of depressive symptoms was assessed using PHQ-9 [39], a concise self-report instrument aligned with the nine diagnostic criteria of DSM-IV. Participants responded to items such as “Little interest or pleasure in doing things” using a scale ranging from 0 (not at all) to 3 (nearly every day). Total scores were obtained by summing responses across all nine items. PHQ-9 demonstrated high internal consistency at both assessment points (α = 0.88) and acceptable test–retest reliability (r = 0.66; see Table 2).

#### 2.2.7. Analytic Strategy

To enable more robust conclusions about potential causal relationships, data were collected across four longitudinal waves. Some participants were absent from individual waves, primarily because of temporary factors such as illness or logistical challenges—such as scheduling conflicts that prevented entire classes or schools from participating. Additionally, the previously mentioned higher attrition among vocational school students further contributed to these gaps in participation. To determine whether these missing data followed a completely random pattern (MCAR), we performed an expectation–maximization (EM) analysis using SPSS Version 27. Little’s MCAR test [40] yielded a significant result, χ^2^(676) = 831.288, *p* < 0.001, indicating that the assumption of data being missing completely at random could not be supported. Since the missing-at-random (MAR) assumption cannot be directly verified—given that it relates to information that was not captured [41]—we used indirect evaluation methods to explore the possibility that these missing data could instead follow a missing-not-at-random (MNAR) pattern. Specifically, we applied a pattern mean difference method [42] and conducted binomial regression analyses to investigate whether dropout or missing responses across measurement waves were systematically associated with depression or mindfulness scores from other time points. To address the issue of multiple comparisons, we employed the Bonferroni correction. None of the eight conducted tests produced statistically significant odds ratios after this adjustment, with *p*-values ranging from 0.035 to 0.82. These findings provided evidence supporting the plausibility that the missing data mechanism was most likely MAR.

Before proceeding with the analyses, we tested longitudinal measurement invariance of both mindfulness measures (CAMM and MASA-A) and the depression measure (PHQ-9) across three levels: configural, metric (weak factorial), and scalar (strong factorial) invariance, utilizing a multiple-group confirmatory factor analysis (CFA) framework. Following the guidelines proposed by Putnick and Bornstein [43], we evaluated model fit using the root mean square error of approximation (RMSEA) and the comparative fit index (CFI). Measurement invariance was considered acceptable if changes in CFI were smaller than 0.01 and changes in RMSEA were less than 0.015 when comparing increasingly constrained models. All three scales in this study were analyzed as unidimensional constructs composed of ordinal items, and the CFA models were estimated using the JASP 0.19.3 statistical software [44].

**Figure 2:**
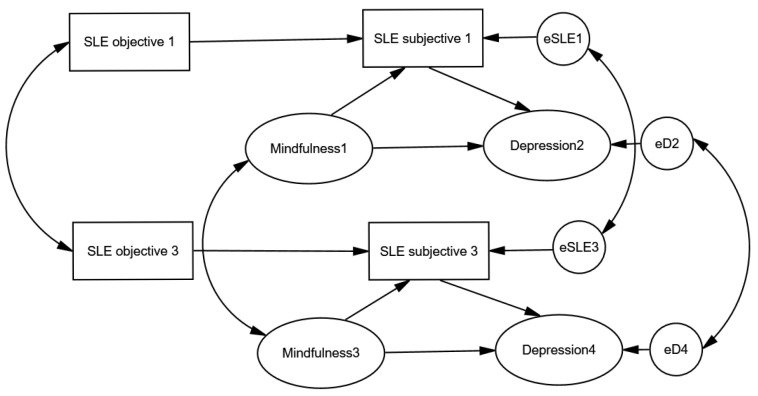
Structural equation model depicting the direct and reappraisal-mediated (indirect) effects of mindfulness on depression.

To explore both the direct and indirect effects of trait mindfulness on depressive symptoms, we tested the full structural model using AMOS [45], as illustrated in Figure 2. We hypothesized that objective stress—defined as the occurrence of a stressful event—would affect the subjective experience of stress. Furthermore, we proposed that cognitive reappraisal, influenced by trait mindfulness, would reduce the perceived severity of stressors. In line with our theoretical framework, mindfulness was expected to mitigate depressive symptoms both indirectly, by facilitating more adaptive stress reappraisal, and directly, through enhanced emotional regulation and reduced rumination. Reappraisal is defined as the hypothetical impact of mindfulness on reducing the subjective perception of a situation’s stressfulness. The core assumption is that perceived stress depends on both the objective level of stress and the cognitive restructuring inherent in mindfulness, which typically lessens perceived adversity. In the proposed model, subjective stress is assumed to be influenced by both objective stress and mindfulness. Reappraisal is therefore operationalized as the portion of variance in subjective stress not accounted for by objective stress, but which can be predicted by mindfulness.

Trait mindfulness and stressful events were assessed concurrently within the same wave, reflecting the assumption that appraisal processes occur simultaneously with the stressful experience. Depressive symptoms were measured in the subsequent wave six months later. To enhance the robustness and generalizability of our findings, we incorporated two consecutive time-lagged sequences within the same model: examining the influence of mindfulness and subjective stress from Wave 1 on depression in Wave 2 and replicating this structure from Wave 3 to Wave 4. This approach enabled cross-validation of the hypothesized effects across distinct time points.

Model fit was evaluated using multiple indices: the χ^2^ goodness-of-fit statistic, which assesses the discrepancy between observed and model-implied covariance matrices, along with the Tucker–Lewis index (TLI), comparative fit index (CFI), and root mean square error of approximation (RMSEA). In line with established guidelines, model fit was considered acceptable if TLI and CFI were close to or exceeded 0.95, and RMSEA was below 0.07 (Hu & Bentler, 1999; Sivo et al., 2006 [46,47]). All analyses were conducted using SPSS Version 27 [48] and AMOS Version 27 [45].

## 3. Results

Prior to initiating the main analyses, we conducted a thorough assessment of longitudinal measurement invariance for all three scales across the study waves. Detailed results, presented in the Supplementary Materials (Tables S2–S4), confirmed that each instrument—CAMM, MAAS-A, and PHQ-9—met the established thresholds for both metric and scalar invariance, supporting the validity of comparisons over time.

Summary statistics for stressful life events (SLEs), depressive symptoms, and mindfulness at each measurement point are provided in Table 2.

An overview of average scores across SLE domains reveals that a considerable proportion of adolescents in both waves experienced at least some stressful events. Stressful events related to relationship problems were the most commonly reported, with an average prevalence of approximately 40%. In contrast, events from the family situation domain were the least frequently reported, with fewer than 20% of adolescents indicating such experiences. However, domain averages should be interpreted with caution, as they may mask variability in the frequency and impact of individual stressors. For instance, everyday stressors such as poor academic performance or the breakup of a close relationship were reported by a large majority, while more severe or less common stressors, such as drug-related issues or the death of a sibling, were rarely endorsed. Despite methodological differences that complicate direct comparisons, these data suggest that Croatian adolescents report a higher frequency of stressful life events compared with their peers in other countries, such as Spain [34]. This pattern extends to depressive symptoms: Croatian adolescents reported significantly higher mean PHQ-9 scores compared with their peers in both Spain (t_1_ = 7.44; t_2_ = 5.10, *p* < 0.001) and Norway (t_1_ = 15.14; t_2_ = 12.94, *p* < 0.001). While the differences with Spain reflect a small effect size (Cohen’s d ranging from 0.18 to 0.25), the differences with Norway indicate a moderate to large effect size (Cohen’s d ranging from 0.52 to 0.57).

These findings may be interpreted in several ways: they could reflect greater exposure to stress and resultant stress-induced depression, a higher vulnerability to depression among Croatian adolescents, or cultural differences in the perception and self-reporting of depressive symptoms. Cross-cultural studies on emotion regulation support the notion that cultural norms influence the internalization and expression of emotional distress, potentially leading to higher self-reported depressive symptoms in cultures where emotional expressivity is more constrained.

Table 3 presents the correlations between mindfulness, depression, and stressful life events (SLEs), with the complete SLE correlation matrix available in the Supplement (Table S1). Depression was consistently and significantly negatively correlated with both mindfulness measures, with correlation coefficients ranging from –0.39 to –0.56. These are substantial associations, particularly given the time interval of six to twelve months between measurements. Additionally, both mindfulness and depression showed low-to-moderate correlations with most SLE domains, including those reflecting more objective experiences (i.e., occurrence of events) and those involving subjective stress appraisal. The only notable exception was the domain related to the loss of loved ones, where correlations were negligible—likely because of limited variability resulting from the low incidence of such events.

**Figure 3:**
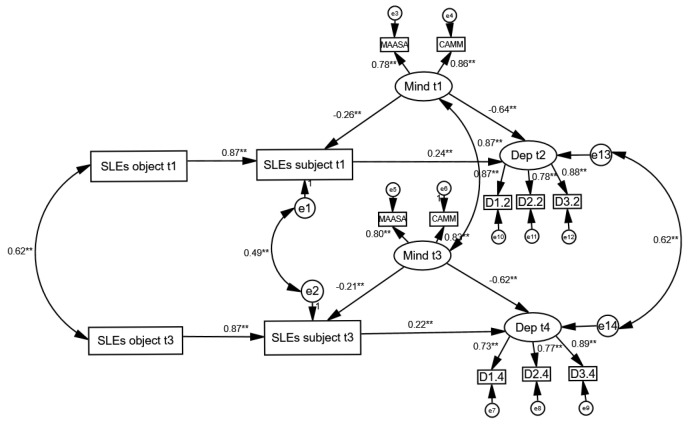
Structural equation model examining the direct and reappraisal-mediated (indirect) effects of mindfulness on depression. Note: The model illustrates the hypothetical direct and indirect reappraisal effects of mindfulness on depression. Depression was assessed at the second time point (t2), six months after the mindfulness and SLEs data were collected (t1). To ensure greater robustness, the same model was also tested in parallel on data from subsequent waves (t3 and t4), one year later. Model fit: χ^2^(69) = 731.997, *p* < 0.001; RMSEA = 0.049 (90% CI 0.046–0.052); CFI = 0.954; TLI = 0.930. The figure displays standardized coefficients. ** *p* < 0.01.

To examine the role of trait mindfulness in alleviating depressive symptoms, we tested the full structural model depicted in Figure 3, using the analysis that included all-encompassing stressful life events (SLEs). The same analytical approach was applied across seven specific domains of SLEs. As the results were largely consistent across these models, with only minor variations, we present a summary of the key model fit indices and path estimates in Table 4. The corresponding structural models are provided in the Supplement (Figures S1–S7).

Table 4 shows that all eight models—seven corresponding to individual SLE domains and one encompassing all SLEs collectively—demonstrated acceptable fit according to the applied model fit criteria. The primary distinction among these models lies in the extent to which mindfulness influences the cognitive reappraisal of stressful situations. Across all models, the effect of trait mindfulness on depression appears to be predominantly direct, with the indirect effect, mediated through reappraisal, being notably weaker. Nonetheless, in each domain, mindfulness consistently contributes to a reduced perception of stress. However, given that stressful events themselves have a relatively limited impact on depressive symptoms, the indirect pathway from mindfulness to depression is negligible in the context of loss or serious illness of a close family member. The strongest, albeit still modest, reappraisal-mediated effect was observed in the domain related to perceived stress from personal health concerns. In other domains, the mediating role of reappraisal is present but remains relatively small.

## 4. Discussion

Previous studies have demonstrated that mindfulness can alleviate depression through two primary pathways: directly, by fostering sustained present-moment awareness and reducing ruminative and negative thought patterns, and indirectly, by promoting cognitive reappraisal of stressful events—encouraging individuals to view them as manageable challenges rather than insurmountable problems. However, much of the existing evidence has been based on cross-sectional research, which limits the ability to draw conclusions about causality. In this study, we employed a longitudinal design, which, by establishing temporal order, provides a stronger basis for inferring causal relationships. Consistent with prior findings, the current study confirms that trait mindfulness exerts a strong direct effect on reducing depressive symptoms, along with a weaker yet consistent indirect effect mediated through reappraisal. While the indirect effect is notably smaller, it remains evident across all examined domains. However, in certain domains, the impact of reappraisal is relatively weak—particularly in cases involving the loss or illness of a close family member, which are commonly cited in the literature as being less amenable to cognitive restructuring. In all other domains, mindfulness exerts a small but discernible effect, which is then indirectly transmitted to depression by shaping stress perception, thereby tending to reduce its intensity.

The findings from our research on a large sample of adolescents contribute to the growing evidence base demonstrating long-term protective effects of mindfulness against depressive symptoms. These effects appear to be mediated through improved stress appraisal and enhanced cognitive coping mechanisms. Consistent with previous longitudinal studies [24,27], our results show that higher levels of trait mindfulness are strongly associated with lower levels of depressive symptoms, with concurrent correlations ranging from −0.60 to −0.70.

Importantly, our structural modeling extends prior work by showing that mindfulness affects depression through two distinct pathways: directly, by fostering more adaptive cognitive responses to stress, and indirectly, though to a lesser extent, by reducing the subjective severity of stress appraisals. This dual-pathway effect aligns with Garland’s Mindfulness to Meaning Theory [31], which suggests that mindfulness enhances emotional regulation through cognitive decentering and positive reappraisal. Our study builds on this framework by applying it across diverse adolescent stress domains, including peer and family relationships, school pressure, and loss, suggesting mindfulness may buffer against depression in a wide range of real-life contexts.

Psychological decentering, as one of the proposed underlying mindfulness pathways, has been found to be an important protective factor in youth mental health. Recent research [49] has shown that the capacity to mentally “step back” from ongoing stress is significantly associated with lower levels of depression and anxiety during adolescence. Therefore, fostering psychological decentering and cognitive reappraisal through mindfulness-based programs not only protects from severe mental health outcomes during adolescence, but potentially lays a beneficial foundation for strengthening executive functioning, thus providing individuals with a more equipped skillset for coping with future stress.

The effectiveness of mindfulness in facilitating adaptive coping is increasingly well-supported, with research advocating for the strong relationship between the two constructs [50]. Mindfulness programs for adolescents and youth have been shown to reduce stress levels and enhance optimism in their own coping abilities [51], along with mitigating numerous mental health outcomes in terms of lowering depressive and anxious symptomatology and fostering psychological well-being [24,52], especially in ethnically diverse and at-risk adolescents [53]. However, the limited availability of developmentally timed and school-based mindfulness interventions remains a significant challenge. Therefore, understanding the underlying mechanisms of change is essential for advocating the integration of such preventive programs into educational settings.

These findings have important implications for school-based mindfulness programs and education policy. Specifically, interventions should emphasize not only general mindfulness practices but also techniques that cultivate emotional regulation and cognitive reappraisal. Embedding these elements into the curriculum could enhance students’ ability to manage stress more effectively and reduce the risk of developing depressive symptoms. Policymakers might also consider incorporating mindfulness training as part of broader mental health strategies within schools, particularly in stress-prone developmental stages such as adolescence.

## 5. Limitations

This study was conducted on a large and mostly representative sample of high school adolescents. Nonetheless, several limitations should be acknowledged. To begin with, the correlational nature of this study prevents firm conclusions about causality. Thus, any causal interpretations should be viewed with caution. Although the longitudinal design strengthens the temporal aspect of the findings, the six-month gaps between measurement points may have been too wide to capture short-term within-person changes or dynamic causal processes. Moreover, this study did not control for additional variables that might influence the observed relationships between stressful life events (SLEs), mindfulness, and depression. Future research could benefit from integrating factors such as coping mechanisms and resilience, which may function as mediators or moderators within the examined relationships. Another noteworthy limitation is this study’s sole reliance on self-reported data for all key constructs, including SLEs, depression, and mindfulness. This approach may introduce various sources of bias, such as social desirability, response tendencies, memory inaccuracies, and common method variance. Common method bias, in particular, can either inflate or deflate the observed relationships between variables, as all measures are collected through the same self-report method. This shared measurement source may have artificially strengthened associations that are, in reality, weaker. To mitigate this limitation and enhance the robustness of future findings, researchers should consider complementing self-reports with alternative assessment methods, such as observer ratings, peer reports, or objective behavioral indicators. Moreover, important individual difference variables—such as personality traits and coping styles—were not examined in the present study but could significantly deepen our understanding of the observed relationships. Therefore, future research that integrates personality, resilience, and coping mechanisms may offer a more comprehensive perspective on the role of mindfulness in adolescent depression.

## 6. Conclusions

Mindfulness has emerged as a promising protective factor in buffering the negative effects of stress and alleviating depressive symptoms among adolescents. However, the exact nature of the relationship between stressful life events, trait mindfulness, and depression remains insufficiently understood. A deeper understanding of this relationship—particularly through longitudinal research—is crucial for uncovering key cognitive mechanisms and guiding the development of effective prevention strategies.

The findings suggest a dual influence of mindfulness on adolescent depression. The more prominent, direct pathway involves the known benefits of mindfulness, such as improved emotional regulation and a reduction in ruminative thinking, which contribute to lower levels of depressive symptoms. The second, less pronounced but still meaningful pathway is indirect: mindfulness facilitates cognitive reappraisal of stressful events, leading to a reduced subjective experience of stress, which in turn lessens its depressive impact.

This research underscores the value of cultivating trait mindfulness, as it emotionally empowers adolescents in two critical ways—by diminishing the perceived impact of stressors and by enhancing their capacity to cope with them. These dual benefits support mindfulness as a preventive tool in reducing the risk of depression during adolescence.

## Figures and Tables

**Table 1:** Demographic composition of the sample across waves.

	N	Biological Sex		Age		School Type	
		% Males	% Females	M	SD	% Vocational	% Gymnasium
Time 1	2486	48.83	51.17	15.91	0.74	60.12	39.88
Time 2	1794	45.54	54.46	16.41	0.71	62.97	37.03
Time 3	2319	45.75	54.25	16.80	0.76	54.91	45.09
Time 4	1575	46.48	53.52	17.38	0.72	53.10	46.90

**Table 2:** Descriptive statistics and reliability indices of measurement instruments.

			M	SD	Min.	Max.	Reliability (Alpha)
Occurrence-based SLEs first wave		Health problems	0.27	0.24	0.00	1.00	0.64
		Bullying, harassment, and abuse	0.33	0.29	0.00	1.00	0.66
		Problems at school	0.26	0.18	0.00	1.00	0.56
		Relationship problems	0.41	0.28	0.00	1.00	0.71
		Loss of loved ones	0.22	0.20	0.00	1.00	0.45
		Health problems of close people	0.33	0.30	0.00	1.00	0.66
		Family situation	0.17	0.18	0.00	1.00	0.72
		TOTAL	0.27	0.16	0.00	1.00	0.87
Severity-based SLEs first wave	Health problems		0.73	0.77	0.00	4.86	0.67
	Bullying, harassment, and abuse		0.76	0.89	0.00	5.00	0.72
	Problems at school		0.62	0.52	0.00	5.00	0.56
	Relationship problems		1.20	1.03	0.00	5.00	0.76
	Loss of loved ones		0.74	0.80	0.00	5.00	0.50
	Health problems of close people		0.98	1.06	0.00	5.00	0.72
	Family situation		0.46	0.58	0.00	5.00	0.76
	TOTAL		0.74	0.55	0.00	4.98	0.89
Occurrence-based SLEs third wave	Health problems		0.26	0.25	0.00	1.00	0.68
	Bullying, harassment, and abuse		0.30	0.30	0.00	1.00	0.70
	Problems at school		0.25	0.19	0.00	1.00	0.63
	Relationship problems		0.37	0.28	0.00	1.00	0.73
	Loss of loved ones		0.19	0.21	0.00	1.00	0.52
	Health problems of close people		0.27	0.30	0.00	1.00	0.70
	Family situation		0.14	0.18	0.00	1.00	0.78
	TOTAL		0.24	0.17	0.00	1.00	0.78
Severity-based SLEs third wave	Health problems		0.72	0.80	0.00	5.00	0.71
	Bullying, harassment, and abuse		0.71	0.89	0.00	5.00	0.73
	Problems at school		0.58	0.55	0.00	5.00	0.66
	Relationship problems		1.09	1.00	0.00	5.00	0.77
	Loss of loved ones		0.62	0.77	0.00	5.00	0.54
	Health problems of close people		0.79	0.99	0.00	5.00	0.73
	Family situation		0.38	0.58	0.00	5.00	0.81
	TOTAL		0.66	0.56	0.00	5.00	0.91
Mindfulness	MAAS-A wave1		3.69	1.00	1.00	6.00	0.90
	MAAS-A wave3		3.70	0.97	1.00	6.00	0.90
	CAMM wave1		2.24	0.84	0.00	4.00	0.87
	CAMM wave3		2.24	0.83	0.00	4.00	0.88
	PHQ-9 depression wave2		9.19	6.31	0.00	27.00	0.88
	PHQ-9 depression wave4		8.70	5.93	0.00	27.00	0.88

**Table 3:** Zero-order correlations among measures of stressful life events, depression, and mindfulness.

		MAASA Wave1	MAASA Wave3	CAMM Wave1	CAMM Wave3	PHQ-9 Wave2	PHQ-9 Wave4
Occurrence-based SLEs first wave	Health problems	−0.360 **	−0.311 **	−0.385 **	−0.339 **	0.454 **	0.361 **
	Bullying, harassment, and abuse	−0.224 **	−0.251 **	−0.306 **	−0.273 **	0.297 **	0.248 **
	Problems at school	−0.126 **	−0.211 **	−0.141 **	−0.154 **	0.212 **	0.202 **
	Relationship problems	−0.230 **	−0.192 **	−0.282 **	−0.199 **	0.261 **	0.252 **
	Loss of loved ones	0.001	−0.114	−0.025	−0.012	0.115 **	0.064
	Health problems of close people	−0.169 **	−0.134 *	−0.261 **	−0.176 **	0.208 **	0.232 **
	Family situation	−0.247 **	−0.167 **	−0.281 **	−0.223 **	0.289 **	0.244 **
	TOTAL	−0.308 **	−0.297 **	−0.377 **	−0.311 **	0.395 **	0.352 **
Severity-based SLEs first wave	Health problems	−0.414 **	−0.359 **	−0.499 **	−0.411 **	0.549 **	0.426 **
	Bullying, harassment, and abuse	−0.311 **	−0.258 **	−0.394 **	−0.341 **	0.383 **	0.332 **
	Problems at school	−0.276 **	−0.263 **	−0.339 **	−0.294 **	0.378 **	0.373 **
	Relationship problems	−0.329 **	−0.220 **	−0.438 **	−0.330 **	0.394 **	0.320 **
	Loss of loved ones	−0.084 *	−0.142 *	−0.125 **	−0.095 **	0.188 **	0.130 **
	Health problems of close people	−0.230 **	−0.146 *	−0.338 **	−0.238 **	0.273 **	0.287 **
	Family situation	−0.291 **	−0.200 **	−0.360 **	−0.299 **	0.339 **	0.314 **
	TOTAL	−0.407 **	−0.324 **	−0.517 **	−0.419 **	0.502 **	0.440 **
Occurrence-based SLEs third wave	Health problems	−0.278 **	−0.329 **	−0.264 **	−0.355 **	0.426 **	0.439 **
	Bullying, harassment, and abuse	−0.196 **	−0.278 **	−0.204 **	−0.307 **	0.399 **	0.325 **
	Problems at school	−0.121 **	−0.145 **	−0.077 *	−0.130 **	0.201 **	0.211 **
	Relationship problems	−0.265 **	−0.217 **	−0.223 **	−0.272 **	0.286 **	0.287 **
	Loss of loved ones	−0.037	−0.085 *	0.046	−0.024	0.137 **	0.045
	Health problems of close people	−0.146 **	−0.203 **	−0.131 **	−0.207 **	0.249 **	0.207 **
	Family situation	−0.189 **	−0.226 **	−0.196 **	−0.266 **	0.322 **	0.280 **
	TOTAL	−0.259 **	−0.322 **	−0.232 **	−0.336 **	0.419 **	0.390 **
Severity-based SLEs third wave	Health problems	−0.329 **	−0.393 **	−0.345 **	−0.436 **	0.530 **	0.524 **
	Bullying, harassment, and abuse	−0.252 **	−0.297 **	−0.287 **	−0.380 **	0.471 **	0.391 **
	Problems at school	−0.176 **	−0.240 **	−0.170 **	−0.274 **	0.344 **	0.323 **
	Relationship problems	−0.342 **	−0.294 **	−0.314 **	−0.408 **	0.413 **	0.408 **
	Loss of loved ones	−0.056	−0.150 **	−0.003	−0.113 **	0.199 **	0.122 **
	Health problems of close people	−0.159 **	−0.236 **	−0.168 **	−0.263 **	0.293 **	0.246 **
	Family situation	−0.204 **	−0.249 **	−0.222 **	−0.341 **	0.385 **	0.337 **
	TOTAL	−0.303 **	−0.388 **	−0.309 **	−0.451 **	0.516 **	0.481 **
Mindfulness	MAASA wave1	-					
	MAASA wave3	0.559 **	-				
	CAMM wave1	0.682 **	0.561 **	-			
	CAMM wave3	0.461 **	0.630 **	0.582 **	-		
	PHQ-9 depression wave2	−0.544 **	−0.547 **	−0.531 **	−0.560 **	-	
	PHQ-9 depression wave4	−0.388 **	−0.531 **	−0.457 **	−0.537 **	0.663 **	-

Note: * *p* < 0.05, ** *p* < 0.01.

**Table 4:** Model Fit Indices and Path Estimates for Structural Models Examining the Direct and Indirect Effects of Mindfulness on Depression.

	χ^2^	df	*p*	TLI	CFI	RMSEA	90% CI	M → SLEs		M → Dep Direct		M → Dep Indirect	
								M1 → SLE1	M3 → SLE3	M1 → Dep2	M3 → Dep4	M1 → Dep2	M3 → Dep4
SLEtot	713.997	69	<0.001	0.930	0.954	0.049	0.046–0.052	−0.259 ***	−0.215 ***	−0.635 ***	−0.621 ***	−0.169 ***	−0.122 ***
SLEhealth	736.994	69	<0.001	0.927	0.952	0.050	0.047–0.053	−0.223 ***	−0.182 ***	−0.625 ***	−0.598 ***	−0.175 ***	−0.124 ***
SLEabuse	552.503	69	<0.001	0.939	0.960	0.042	0.039–0.046	−0.177 ***	−0.168 ***	−0.701 ***	−0.657 ***	−0.075 ***	−0.062 ***
SLEschool	272.490	69	<0.001	0.972	0.982	0.028	0.024–0.031	−0.278 ***	−0.205 ***	−0.705 ***	−0.679 ***	−0.041 ***	−0.030 ***
SLErelations	471.399	69	<0.001	0.952	0.969	0.039	0.035–0.042	−0.252 ***	−0.231 ***	−0.687 ***	−0.656 ***	−0.037 ***	−0.030 ***
SLEloss	171.525	69	<0.001	0.988	0.992	0.020	0.016–0.023	−0.124 ***	−0.107 ***	−0.739 ***	−0.702 ***	−0.022	−0.018
SLEhealth-close	375.590	69	<0.001	0.965	0.977	0.034	0.030–0.037	−0.131 ***	−0.098 ***	−0.734 ***	−0.690 ***	−0.008	−0.009
SLEfamily	480.769	69	<0.001	0.953	0.969	0.039	0.036–0.042	−0.140 ***	−0.126 ***	−0.708 ***	−0.672 ***	−0.019 ***	−0.016 ***

Note. SLE = Stressful Life Events. SLE domains include: Health Issues, Harassment and Abuse, School, Relationships, Loss of a Close Person, Health Issues of a Close Person, and Family. M → SLEs = Path estimates representing the effect of mindfulness on the reappraisal of stressful events; M → Dep (direct) = Direct path estimates of mindfulness effects on depression; M → Dep (indirect) = Indirect path estimates of mindfulness effects on depression through reappraisal. *** *p* < 0.001.

## Data Availability

The raw data supporting the conclusions of this article will be made available by the authors on request.

## Supplementary Materials

The following supporting information can be downloaded at: https://www.mdpi.com/article/10.3390/medicina61071154/s1, Figure S1: Structural Equation Model Examining the Direct Effects of Mindfulness on Depression and the Indirect Effects Mediated by Reappraisal of Stressful Health-Related Events title; Figure S2: Structural Equation Model Examining the Direct Effects of Mindfulness on Depression and the Indirect Effects Mediated by Reappraisal of Stressful Harassment and Abuse-Related Events; Figure S3. Structural Equation Model Examining the Direct Effects of Mindfulness on Depression and the Indirect Effects Mediated by Reappraisal of Stressful School-Related Events; Figure S4. Structural Equation Model Examining the Direct Effects of Mindfulness on Depression and the Indirect Effects Mediated by Reappraisal of Stressful Relationship-Related Events; Figure S5. Structural Equation Model Examining the Direct Effects of Mindfulness on Depression and the Indirect Effects Mediated by Reappraisal of Stressful Events Related to the Loss of a Close Person; Figure S6. Structural Equation Model Examining the Direct Effects of Mindfulness on Depression and the Indirect Effects Mediated by Reappraisal of Stressful Events Related to the Health Issues of a Close Person; Figure S7. Structural Equation Model Examining the Direct Effects of Mindfulness on Depression and the Indirect Effects Mediated by Reappraisal of Stressful Family-Related Events; Table S1. Zero-Order Correlations Between Measures of Stressful Life Events at Two Time Points; Table S2. Longitudinal Measurement Invariance Results for the Mindfulness Scale (CAMM); Table S3. Longitudinal Measurement Invariance Results for the Mindfulness Scale (MAAS-A); Table S4. Longitudinal Measurement Invariance Results for the Depression Scale (PHQ-9).
