# Supplementary material for: Mindfulness Reduces Adolescent Depression Through Stress Appraisal and Cognitive Reactivity: Evidence from a Four-Wave Longitudinal Study
Source: Medicina (Kaunas). 2025 Jun 26;61(7):1154. doi: 10.3390/medicina61071154 (PMC12299247; doi:10.3390/medicina61071154)
Supplement: Supplementary file 1 [file medicina-61-01154-s001.zip › medicina-3703681-supplementary.pdf]

Table S1. *Zero-Order Correlations Between Measures of Stressful Life Events at Two Time Points*

			1	2	3	4	5	6	7	8	9	10	11	12	13	14	15	16	17	18	19	20	21	22	23	24	25	26	27
Occurrence-based SLE first wave	1	Health problems	--																										
	2	Bullying, harassment and abuse	.45	--																									
	3	Problems at school	.35	.33	--																								
	4	Relationship problems	.44	.42	.45	--																							
	5	Loss of loved ones	.20	.19	.30	.29	--																						
	6	Health problems of close people	.39	.38	.29	.37	.38	--																					
	7	Family situation	.42	.39	.35	.42	.28	.47	--																				
Severity-based SLE first wave	8	Health problems	.89	.44	.29	.41	.17	.38	.41	--																			
	9	Bullying, harassment and abuse	.46	.85	.27	.38	.16	.36	.39	.53	--																		
	10	Problems at school	.39	.35	.80	.40	.24	.31	.33	.44	.41	--																	
	11	Relationship problems	.47	.44	.39	.86	.23	.37	.40	.53	.50	.49	--																
	12	Loss of loved ones	.20	.20	.28	.29	.90	.39	.26	.23	.23	.31	.31	--															
	13	Health problems of close people	.36	.36	.26	.35	.36	.90	.43	.42	.42	.36	.43	.46	--														
	14	Family situation	.39	.37	.30	.40	.25	.45	.90	.46	.44	.38	.47	.30	.50	--													
Occurrence-based third wave	15	Health problems	.53	.28	.24	.31	.15	.20	.27	.52	.31	.30	.35	.18	.20	.28	--												
	16	Bullying, harassment and abuse	.30	.48	.26	.30	.14	.19	.27	.32	.47	.30	.32	.16	.21	.29	.48	--											
	17	Problems at school	.23	.21	.43	.31	.17	.12	.22	.18	.18	.32	.26	.14	.10	.20	.40	.42	--										
	18	Relationship problems	.35	.32	.37	.57	.16	.19	.33	.32	.30	.32	.54	.19	.19	.31	.44	.46	.47	--									
	19	Loss of loved ones	.13	.12	.21	.24	.38	.16	.19	.09	.10	.13	.17	.36	.16	.15	.30	.27	.42	.39	--								
	20	Health problems of close people	.29	.23	.18	.25	.26	.39	.30	.29	.23	.18	.25	.26	.39	.30	.36	.37	.35	.38	.45	--							
	21	Family situation	.31	.31	.25	.29	.13	.22	.54	.31	.31	.19	.29	.14	.22	.53	.44	.42	.46	.44	.42	.47	--						
Severity-based third wave	22	Health problems	.51	.29	.21	.28	.11	.22	.29	.58	.36	.32	.38	.16	.25	.36	.90	.47	.36	.42	.25	.35	.43	--					
	23	Bullying, harassment and abuse	.30	.44	.22	.29	.12	.22	.29	.37	.53	.33	.37	.16	.27	.37	.47	.85	.35	.41	.22	.34	.40	.56	--				
	24	Problems at school	.24	.23	.35	.28	.13	.14	.23	.25	.26	.39	.31	.14	.17	.28	.44	.44	.82	.44	.38	.35	.48	.49	.49	--			
	25	Relationship problems	.35	.34	.31	.52	.13	.21	.32	.38	.37	.35	.60	.20	.25	.36	.43	.46	.39	.87	.30	.38	.42	.50	.51	.49	--		
	26	Loss of loved ones	.12	.13	.19	.24	.37	.19	.19	.12	.14	.16	.22	.40	.22	.20	.28	.26	.37	.38	.90	.44	.38	.30	.26	.41	.39	--	
	27	Health problems of close people	.27	.23	.16	.24	.22	.38	.29	.31	.26	.19	.27	.26	.44	.34	.34	.34	.30	.35	.40	.91	.42	.40	.38	.37	.44	.47	--
	28	Family situation	.28	.28	.19	.27	.09	.20	.51	.32	.31	.18	.32	.12	.23	.57	.42	.40	.40	.42	.36	.45	.89	.50	.47	.53	.51	.42	.51

Table S2. *Longitudinal Measurement Invariance Results for the Mindfulness Scale (CAMM)*

	Model	χ^2	df	p	CFI	RMSEA	$\Delta\chi^2$	df	p	ΔCFI	ΔRMSEA	Invariance
	Configural	2983.655	70	< .001	0.924	0.146						
First wave	Metric	1808.337	79	< .001	0.955	0.106	-1175.318	9	.002	-0.031	-0.040	Yes
	Scalar	2244.037	108	< .001	0.944	0.101	435.7	29	< .001	0.011	-0.005	Yes

Note. df = degrees of freedom; CFI = comparative fit index; RMSEA = root-mean-square error of approximation.

Table S3. *Longitudinal Measurement Invariance Results for the Mindfulness Scale (MAAS-A)*

	Model	χ^2	df	p	CFI	RMSEA	$\Delta\chi^2$	df	p	ΔCFI	ΔRMSEA	Invariance
	Configural	1349.827	154	< .001	0.941	0.101						
First wave	Metric	947.154	167	< .001	0.961	0.078	-402.673	13	.002	-0.020	-0.023	Yes
	Scalar	1138.937	222	< .001	0.954	0.073	191.783	55	< .001	0.007	-0.005	Yes

Note. df = degrees of freedom; CFI = comparative fit index; RMSEA = root-mean-square error of approximation.

Table S4. *Longitudinal Measurement Invariance Results for the Depression Scale (PHQ-9)*

	Model	χ^2	df	p	CFI	RMSEA	$\Delta\chi^2$	df	p	ΔCFI	ΔRMSEA	Invariance
	Configural	664.473	54	< .001	0.972	0.090						
First wave	Metric	461.802	62	< .001	0.982	0.068	-202.671	8	.003	-0.010	-0.022	Yes
	Scalar	559.96	79	< .001	0.978	0.066	98.158	17	.030	0.004	-0.002	Yes

Note. df = degrees of freedom; CFI = comparative fit index; RMSEA = root-mean-square error of approximation.

Figure S1. Structural Equation Model Examining the Direct Effects of Mindfulness on Depression and the Indirect Effects Mediated by Reappraisal of Stressful **Health-Related Events**

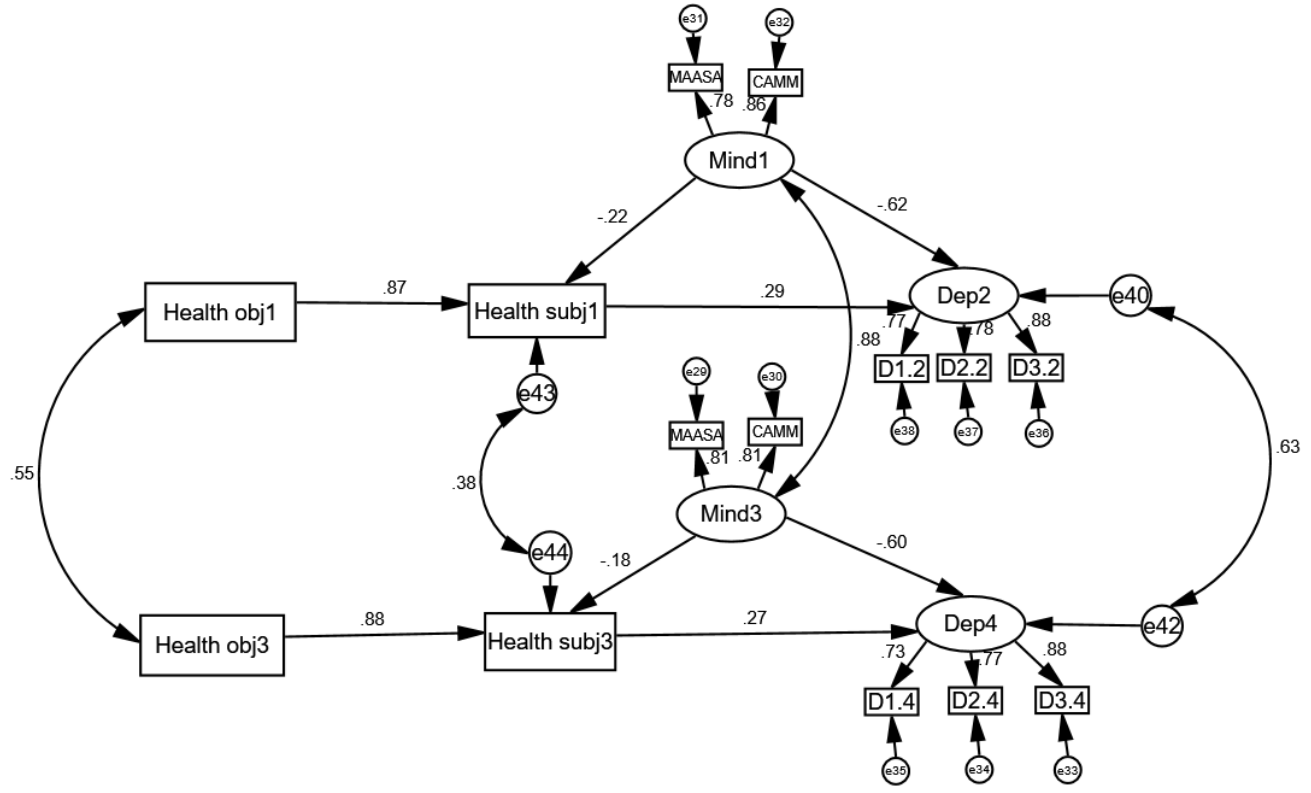

Note: Model fit: $\chi^2(69) = 736.994$, $p < .001$; RMSEA = .050 (90% CI .047 - .053); CFI = .952; TLI =0.927. The figure displays standardized coefficients.

Figure S2. Structural Equation Model Examining the Direct Effects of Mindfulness on Depression and the Indirect Effects Mediated by Reappraisal of Stressful **Harassment and Abuse-Related Events**

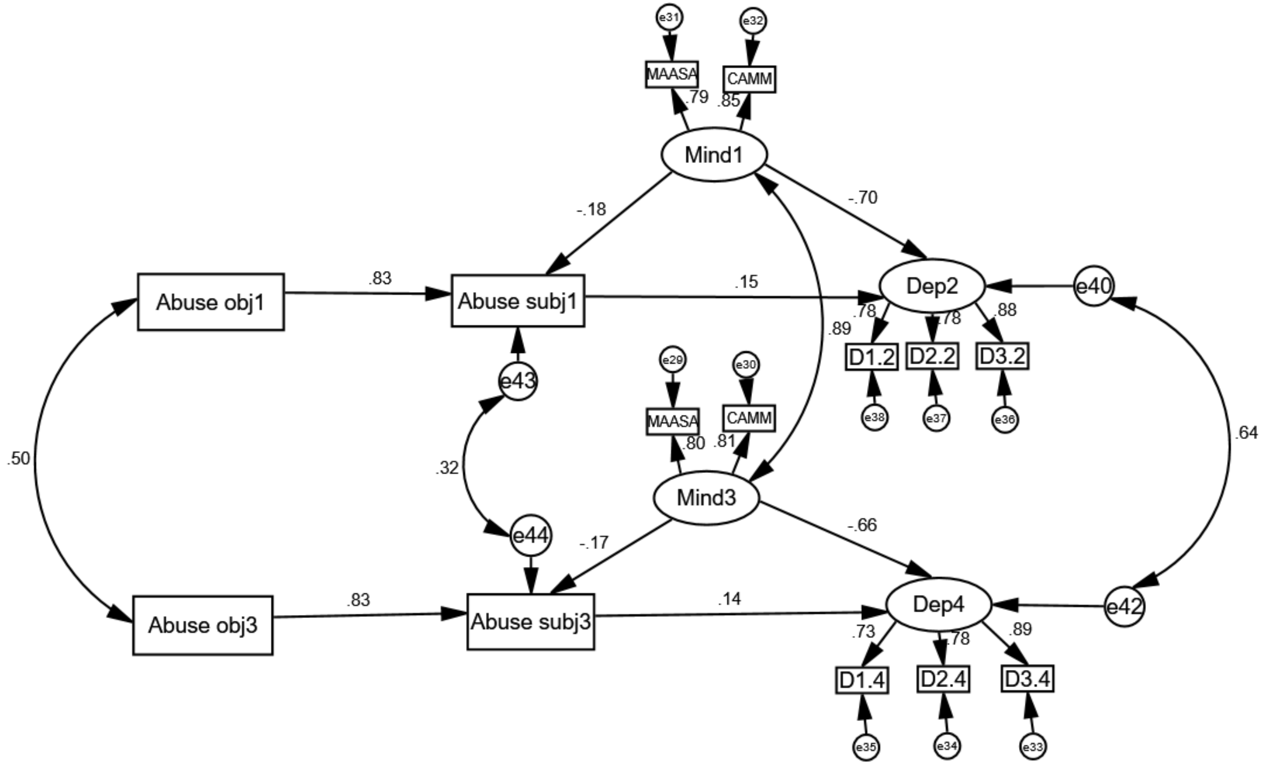

Note: Model fit: $\chi^2(69) = 552.503$, $p < .001$; RMSEA = .042 (90% CI .039 - .046); CFI = .960; TLI =0.939. The figure displays standardized coefficients.

Figure S3. Structural Equation Model Examining the Direct Effects of Mindfulness on Depression and the Indirect Effects Mediated by Reappraisal of Stressful **School-Related Events**

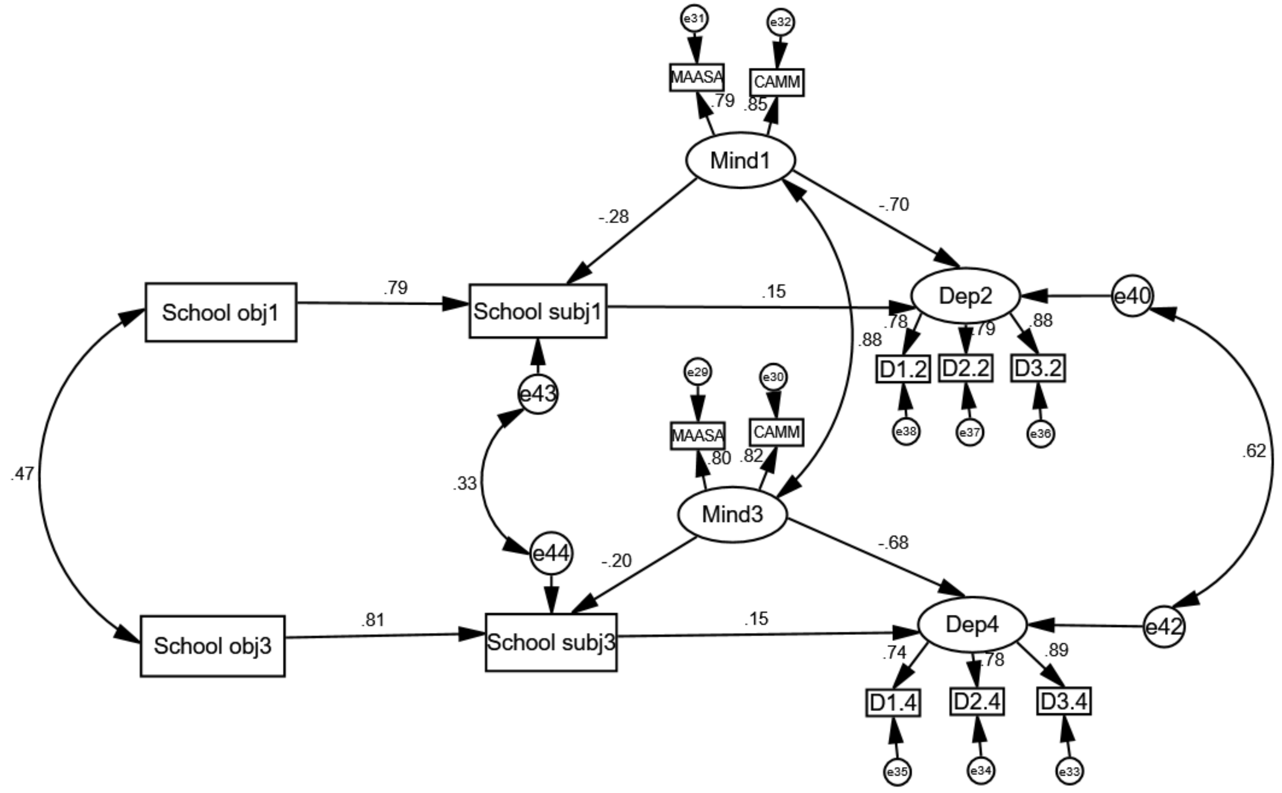

Note: Model fit: $\chi^2(69)$ 272.49, $p < .001$; RMSEA = .028 (90% CI .024 - .031); CFI = .982; TLI =0.972. The figure displays standardized coefficients.

Figure S4. Structural Equation Model Examining the Direct Effects of Mindfulness on Depression and the Indirect Effects Mediated by Reappraisal of Stressful **Relationship-Related Events**

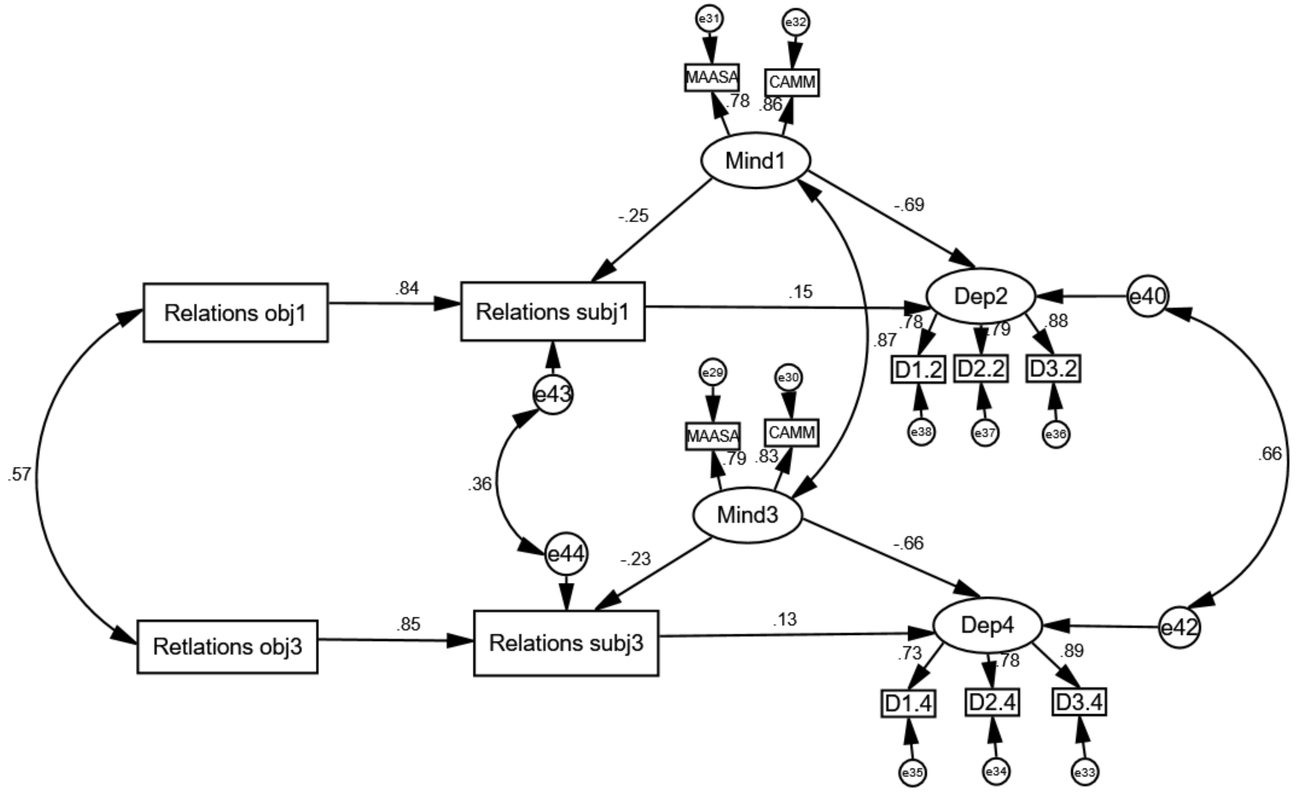

Note: Model fit: $\chi^2(69) = 471.399$, $p < .001$; RMSEA = .039 (90% CI .035 - .042); CFI = .969; TLI =0.952. The figure displays standardized coefficients.

Figure S5. Structural Equation Model Examining the Direct Effects of Mindfulness on Depression and the Indirect Effects Mediated by Reappraisal of **Stressful Events Related to the Loss of a Close Person**

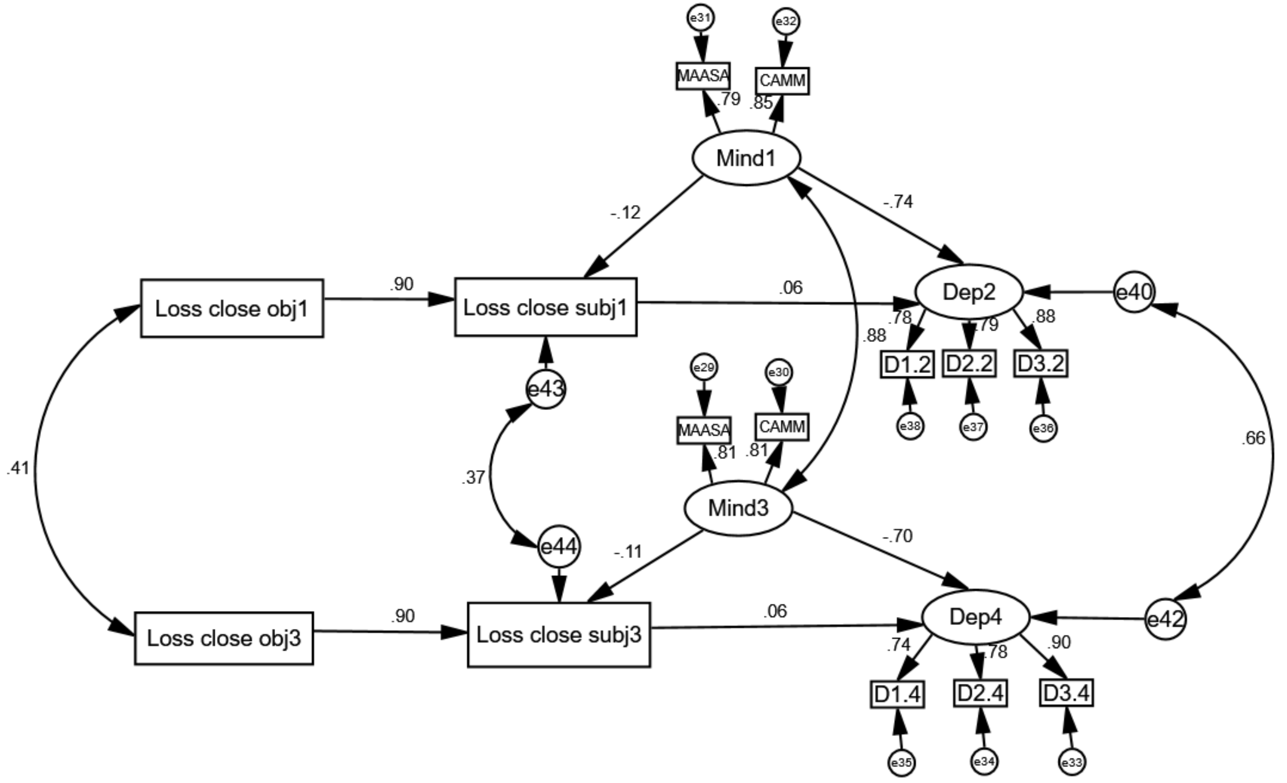

Note: Model fit: $\chi^2(69) = 171.525$, $p < .001$; RMSEA = .020 (90% CI .016 - .023); CFI = .992; TLI =0.988. The figure displays standardized coefficients.

Figure S6. Structural Equation Model Examining the Direct Effects of Mindfulness on Depression and the Indirect Effects Mediated by Reappraisal of **Stressful Events Related to the Health Issues of a Close Person**

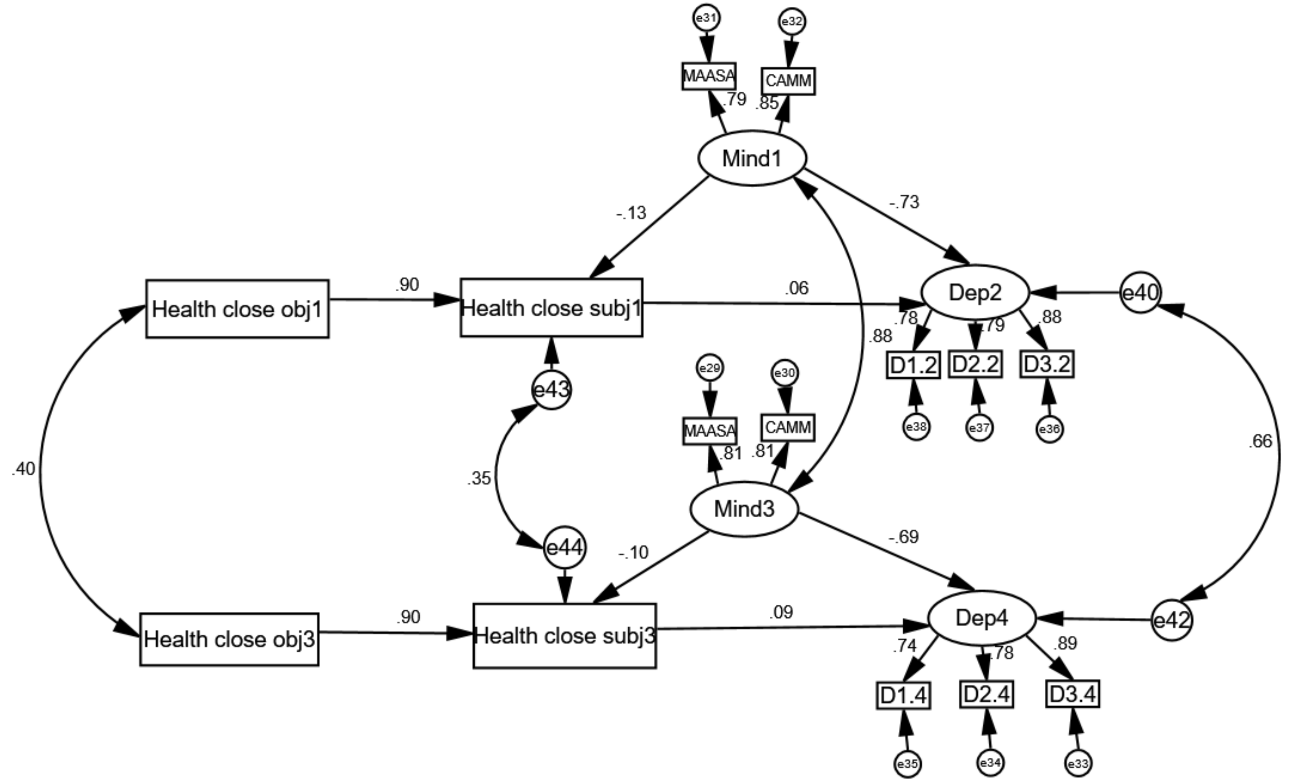

Note: Model fit: $\chi^2(69) = 375.590$, $p < .001$; RMSEA = .034 (90% CI .030 - .037); CFI = .977; TLI =0.965. The figure displays standardized coefficients.

Figure S7. Structural Equation Model Examining the Direct Effects of Mindfulness on Depression and the Indirect Effects Mediated by Reappraisal of **Stressful Family-Related Events**

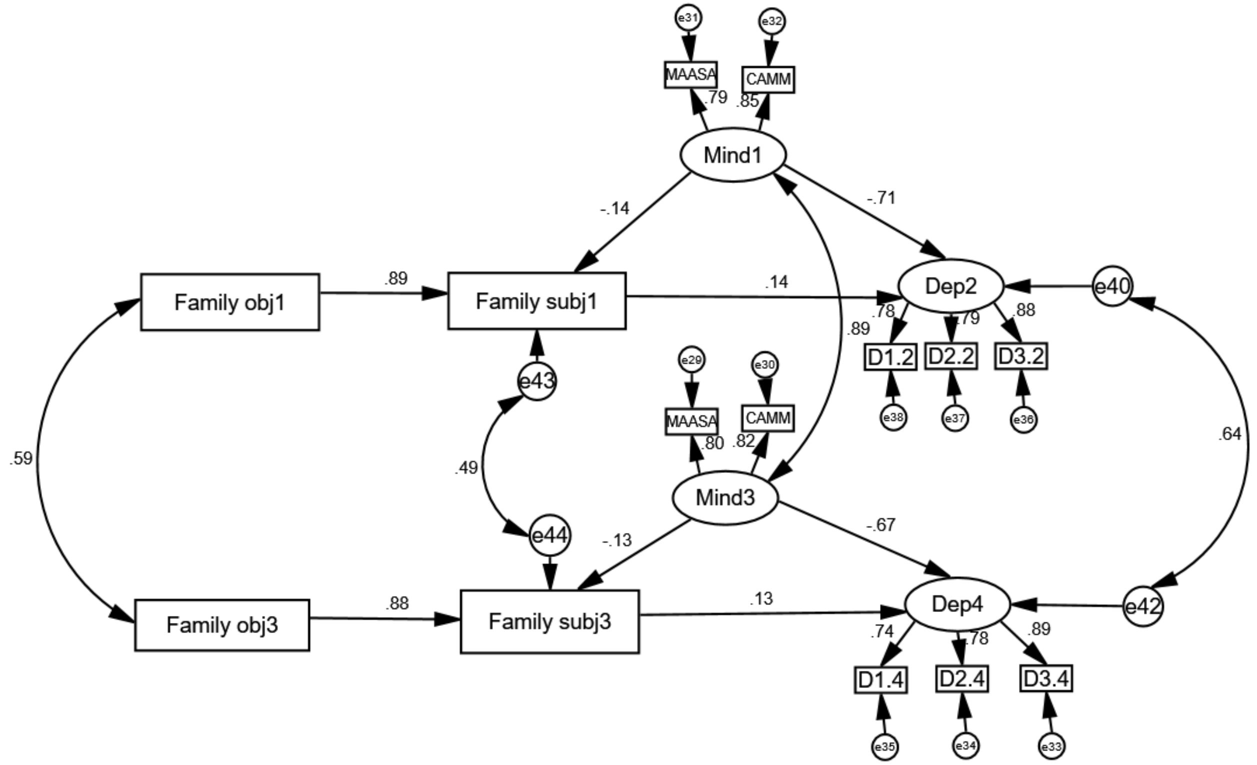

Note: Model fit: $\chi^2(69) = 480.769$, $p < .001$; RMSEA = .039 (90% CI .036 - .042); CFI = .969; TLI =0.953. The figure displays standardized coefficients.
